# A Working-Class View of Our Hospitals

**Published:** 1888-01-14

**Authors:** A. W. Webster

**Affiliations:** Chairman of Workmen's Governors, Sunderland Infirmary


*(To be continued.)*

### A Working-Class View of Our Hospitals.

By A. W. Webster, Chairman of Workmen's Governors, Sunderland Infirmary. (*Continued from p.* 258.)

Very few hospital managers need to be told that care and attention on the part of the nurses, coupled with skilful medical supervision from the honorary staff, is absolutely necessary to ensure efficiency. Where the one or the other is defective you will certainly fail to popularise the institution among the workmen who use it. I daresay I have no reason for hiding the fact that I have selected Sunderland Infirmary as my model, and although there may yet be considerable room for improvement, I think those who know it best will readily admit all I claim on its behalf, and those who know nothing of its existence may benefit by my rehearsal of its leading features. Any visitor to Sunderland caring to make the slightest inquiry, will soon find undoubted proofs of the popularity of the local infirmary among all classes of the community. I think I am strictly within the truth when I say that its popularity may be dated from the day it was declared a free hospital. The system of representation then introduced enabled the workmen to share in the management, and an increased interest in the institution has been the result. The workmen's subscriptions have become what they ought to be, a vital and necessary part of its income, reaching nearly fifty per cent. of the total last year. To say that our workmen are interested in the infirmary is doing them but scant justice. Large numbers of them believe it to be a veritable Bethesda in their midst, and are prepared to make considerable sacrifice to support and maintain its efficiency.

Perhaps the testimony of a patient (being one of many such continually received by some of our officials) will be the best proof of our condition. He says in a letter to me a few weeks ago: "The medical treatment, general arrangement, cleanliness, and ventilation are perfect. If I refer at all to the sisters (nurses) it must be in terms of the highest praise. Their whole lives are devoted to the amelioration of pain. They seem to me to be boldest when it means personal risk to themselves alone."

Reference to this infirmary is seldom made without bestowing unlimited praise on the sisters or nurses. These ladies possess all the heroism so nobly displayed by Florence Nightingale. They have voluntarily given up the comforts and advantages of social life, and after going through a course of preparation in the Protestant Evangelical Training Institution at Tottenham, are now to be found exemplifying Christianity in its most practical shape by tending and caring for the poor and suffering. I need simply add that it is this constant self-sacrificing zeal of the nurses, together with the devoted and skilled attention of the honorary medical staff and the broad and liberal management of the committee, that have endeared this institution to the heart of all within the radius of its influence.

Before commencing with a detailed account of the successful manner in which our workmen's subscriptions have been organised and developed, I must pay a tribute of respect and praise to the indefatigable energy and tact of Mr. Thomas Robinson, the Infirmary Secretary, whose remarkable aptitude and great ability have contributed largely to the present success. For the purpose of comparison I have tried to glean particulars of the systems and schemes adopted by the workpeople's agencies in other towns, and I find the general practice to be that of having more or less representative committees of workmen, whose aim is chiefly to direct funds by and through their agency to the local charities. Some organise galas, fêtes, etc., others assist with Hospital Saturday collections, and some even go from house to house soliciting subscriptions to further the end in view. These are all no doubt desirable and commendable means of operation, yet they lack the foundation principle of direct support from the great mass of the working class. It is simply another channel formed through which the donations of all classes, rich and poor, can flow into the hospital funds.

A very generally expressed opinion among Christian workers is, that if the one talent Christians could be got to realise the power of their single talent and put it out to usury, it would soon revolutionise the Church life of Christendom. We have discovered by experience that a similar revolutionising power rests in the workmen's pence in reference to hospital management, and after a fair amount of work in both spheres, I have little hesitation in proclaiming that the workmen's "penny" is much more easily converted to activity than the dormant and rusty talent possessed by so many who profess to follow the Master. The best givers to every good work, be they rich or poor, are those who subscribe systematically and regularly; and the whole success of our workmen's efforts lies in the fact that they appreciate and act on this principle. Nearly every horny-handed artisan and labourer in and around our town contribute their penny per week to Sunderland Infirmary as regularly as they receive their wages.

